# Molecular Targeting of the Phosphoinositide-3-Protein Kinase (PI3K) Pathway across Various Cancers

**DOI:** 10.3390/ijms25041973

**Published:** 2024-02-06

**Authors:** Khine S. Shan, Amalia Bonano-Rios, Nyein Wint Yee Theik, Atif Hussein, Marcelo Blaya

**Affiliations:** 1 Division of Hematology and Oncology, Memorial Health Care, Pembroke Pines, FL 33028, USA; abonanorios@mhs.net (A.B.-R.); ahussein@mhs.net (A.H.); mblaya@mhs.net (M.B.); 2 Division of Internal Medicine, Memorial Health Care, Pembroke Pines, FL 33028, USA; ntheik@mhs.net

**Keywords:** phosphatidylinositol-3-kinase, PI3K, AKT, mTOR, targeted therapy, molecular profiling, personalized medicine

## Abstract

The dysregulation of the phosphatidylinositol-3-kinase (PI3K) pathway can lead to uncontrolled cellular growth and tumorigenesis. Targeting PI3K and its downstream substrates has been shown to be effective in preclinical studies and phase III trials with the approval of several PI3K pathway inhibitors by the Food and Drug Administration (FDA) over the past decade. However, the limited clinical efficacy of these inhibitors, intolerable toxicities, and acquired resistances limit the clinical application of PI3K inhibitors. This review discusses the PI3K signaling pathway, alterations in the PI3K pathway causing carcinogenesis, current and novel PI3K pathway inhibitors, adverse effects, resistance mechanisms, challenging issues, and future directions of PI3K pathway inhibitors.

## 1. Introduction

Phosphatidylinositol-3-kinase or phosphoinositide-3-protein kinase (PI3K)/AKT (protein kinase B)/mammalian target of rapamycin (mTOR) signaling pathway represents one of the most essential intracellular signaling pathways that regulate cellular transcription, translation, proliferation, growth, survival, metabolism, and angiogenesis [1]. The activation of the PI3K/AKT/mTOR pathway plays an important role in the development of various cancers and resistance to cancer treatments [2]. The PI3K/AKT/mTOR pathway dysregulation is considered one of the most mutated pathways in cancers and has been found across all human cancers including hematological malignancies, breast, lung, head and neck, glioblastomas, prostate, and gastrointestinal cancers; thus, targeting individual components in this pathway (PI3K, AKT, and mTOR) may be a potential therapeutic treatment for various malignancies [3]. Therefore, it is crucial to detect PI3K pathway gene alterations as it can provide a practical therapeutic approach. Tracking the evolution of PI3K alterations during treatment can provide physicians with information to adapt treatment strategies and overcome resistance mechanisms [4]. The detection of gene alterations in this pathway has been revolutionized in clinical oncology by directing therapeutic decisions, improving in terms of prognostic value, and facilitating the development of additional therapies.

Small molecule inhibitors of PI3K include pan-PI3K inhibitors, isoform-selective PI3K inhibitors, and PI3K/mTOR inhibitors [5]. The PI3K inhibitors (PI3KIs) have been investigated in numerous preclinical and clinical trials and have proven to be effective in slowing tumor progression. The inhibitors of downstream substrates AKT and mTOR have also been investigated. However, the clinical efficacy of these inhibitors as monotherapies is so far limited because the PI3K pathway is activated via diverse and redundant ways. PI3K pathway activation is associated with resistance to other therapies; therefore, PI3K pathway inhibitors in combination with other drugs could revive the therapeutic sensitivity to these drugs. For instance, mTOR inhibitors can lead to AKT phosphorylation via paradoxical feedback activation leading to the resistance of mTOR inhibitors. PI3K inhibition has been shown to decrease AKT phosphorylation following mTOR inhibition [6,7]. Idelalisib is the first PI3KI approved by the United States Food and Drug Administration (FDA) in July 2014, for the treatment of relapsed chronic lymphocytic leukemia (CLL), relapsed small lymphocytic lymphoma (SLL), and relapsed follicular lymphoma (FL) [8,9].

In recent years, there have been more PI3KIs approved in cancer treatments, however intrinsic, and extrinsic resistance as well as adverse effects limit the clinical application of PI3KIs. In this review, we will discuss the PI3K signaling pathway, its alterations across various cancers, and the current FDA-approved PI3KIs as well as other PI3KIs under investigation. We will also briefly cover AKT and mTOR inhibitors, which are inhibitors of downstream substrates in the PI3K pathway. We will then review the limitations of therapeutic PI3KIs due to their toxicities and mechanisms of resistance as well as the current ongoing clinical trials and potential directions of PI3K pathway inhibitors.

## 2. PI3K/AKT/mTOR Pathway

The PI3K signaling pathway is a complex series of events initiated at the cellular membrane level, involving tightly regulated mechanisms and involves PI3K, AKT, and mTOR substrates. PI3Ks are divided into three different classes (I-II-II) depending on their structural and specific features [10]. Class I PI3Ks are again subclassified into class IA (PI3Kα, PI3Kβ, and PI3Kδ) and class IB (PI3Kγ) encoded by *PIK3CA*, *PIK3CB*, *PIK3CD* and *PIK3CG* genes, respectively, consisting of a catalytic p110 subunits (p110α, p110β, p110δ, or p110γ) and a regulatory subunit [11,12]. The catalytic subunit of class I PI3K is accompanied by a regulatory subunit, and in its inactive state, the two subunits form an inactive heterodimer complex located in the cytosol. Each of these four class I PI3K catalytic isoforms mediates signal transduction and tumor cell survival based on the type of cancer and the genetic or epigenetic variations it harbors [12]. For instance, p110α is important in the signaling and tumor growth driven by *PIK3CA* mutations and/or oncogenic receptor tyrosine kinases, whereas p110β isoform is essential in mediating tumorigenesis arising from PTEN loss [12]. On the other hand, the p110δ isoform is predominantly expressed in leukocytes and important for the survival and function of regulatory T cells (Treg) and myeloid-derived suppressor cells, making it an important target for the inhibition in the treatment of hematologic malignancies [12,13].

Class II PI3Ks play an essential role in cellular processes including cell growth, migration, survival, metabolism, endocytosis, vesicular trafficking, and angiogenesis [14]. Class II PI3Ks consist of three catalytic isoforms PI3KC2α, PI3KC2β, and PI3KC2γ which are encoded by the *PIK3C2A*, *PIK3C2B*, and *PIK3C2G* genes, respectively, without any regulatory subunits and can regulate the internalization of receptors [14,15,16,17]. Class III PI3Ks are the heterodimers with a catalytic subunit of VPS34 encoded by *PIK3C3* and a regulatory and accessory subunit of VPS15 encoded by *PIK3R4* [14,17]. Class III PI3Ks are important for autophagy, endosomal trafficking, macrophage phagocytosis, activation of signal transduction, and modulation of different protein kinases [14].

AKT, also known as protein kinase B, is a serine and threonine kinase that cleaves phospholipids on serine and threonine residues of target proteins. There are three different isoforms of AKT: AKT1 (PKBα), AKT2 (PKBβ), and AKT3 (PKBγ), each with distinct expression patterns and functions in different tissues [3]. AKT plays a crucial role in cell survival and proliferation by regulating various cellular processes, including glucose uptake and utilization, glycogen and protein synthesis, cell survival and proliferation, and fatty acid synthesis. AKT has three key regulatory sites, including a PH (pleckstrin homology) domain, threonine 309, and serine 473, which are critical for its activation and function [18]. mTOR is a protein kinase that serves as the catalytic subunit of two multi-protein complexes such as mTOR complex 1 (mTORC1) and mTOR complex 2 (mTORC2), which regulate cell growth [19].

The activation of the PI3K protein is initiated via the activation of transmembrane receptors, such as Receptor Tyrosine Kinase (RTK), including epidermal growth factor receptor (EGFR)/ERBB, fibroblast growth factor receptor (FGFR), platelet-derived growth factor receptor (PDGFR), insulin receptor (INSR), or G-Protein coupled receptor (GPCR), or through RAS (Rat Sarcoma)-mediated proteins [20]. In normal cells, a ligand binds to a cellular membrane RTK, leading to its activation via the dimerization and autophosphorylation of these molecules. This subsequently leads to the interaction of the RTK with the SH2 (Src Homology 2) domain of the p85 regulatory subunit, which forms a heterodimer with the p110 subunit and activates it [21,22]. PI3K can also be activated through direct interaction with RAS-binding proteins (RBD) located in p110. The PI3Kγ isoform can be activated via RAS-dependent mechanisms, and PI3K can be directly activated by RAS-GTP [19].

**Figure 1:**
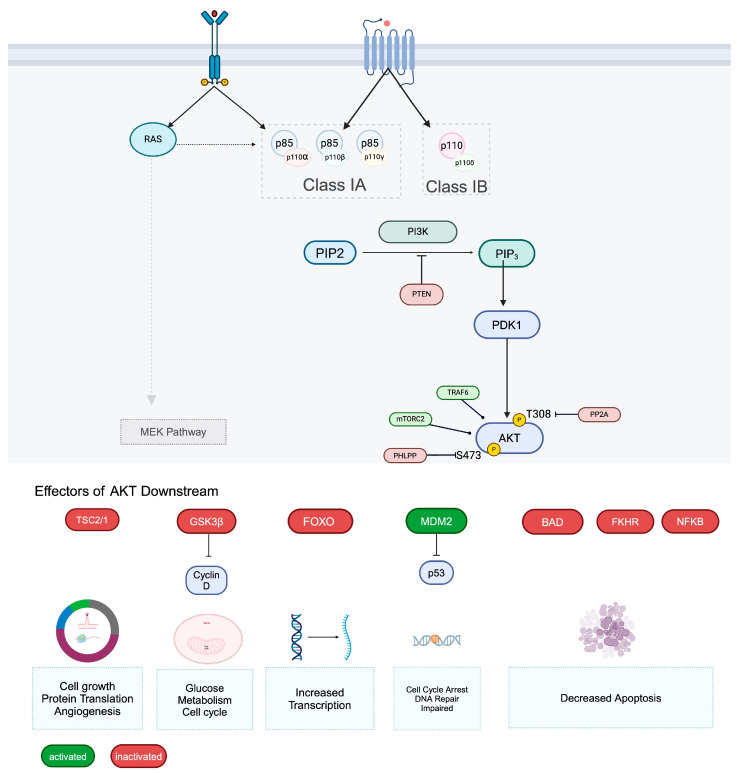
A schematic representation of the PI3K/AKT signaling pathway and its activation. This pathway can be activated by various extracellular stimuli, including the GTP-mediated binding complex induced by chemokines or RTK-mediated complex (GF, EGF, FGF, etc.). Activated PI3K phosphorylates PIP2 to PIP3, a process that is inhibited by PTEN. PIP3 then activates PDK1, which in turn activates AKT. AKT contains two binding domains at S474, which can be inhibited by PHLPP, and T308, which can be inhibited by PP2A. Some known stimulators of AKT include TRAP4 and mTORC2. Activated AKT contains over 100 effector molecules and is directly involved in cell growth arrest, increased protein translation, increased transcription, impaired DNA repair mechanisms, decreased apoptosis, and increased glucose metabolism. Created with BioRender.com.

Activated PI3K then phosphorylates phosphatidylinositol 4,5-bisphosphate (PIP2) at the plasma membrane to phosphatidylinositol (3,4,5)-trisphosphate (PIP3). Increased PIP3 concentration at the plasma membrane leads to the recruitment of proteins containing PIP3 binding PH domains such as PDK1 (Pyruvate Dehydrogenase Kinase 1) and AKT kinases [19]. PDK1 contains a PH domain that has a high affinity for PIP3 and becomes active upon binding PIP3 to its PH domain, leading to the phosphorylation at the serine 241 residue, and the activation of AKT via phosphorylation at the threonine 389 residue [2]. Additionally, PDK1 can indirectly activate AKT via the activation of mTORC2, which activates AKT by binding to the serine 473 residue [18]. Activated AKT then phosphorylates downstream effector proteins including mTOR complexes as well as FOXO (Forkhead box) transcription factors that regulate cell survival and cell cycle progression [19,20]. However, the duration of PIP3 activity is short due to the antagonizing mechanism by PTEN and other lipid phosphatases [19]. Figure 1 summarizes the PI3K pathway activation.

## 3. Role of PI3K Pathway Alterations across Various Cancers

The dysregulation of proteins in the PI3K pathway can cause the development of cancers. Among the three classes of PI3Ks, class I PI3Ks are of particular interest and the most investigated given their important role in the development of human malignancies. Amplifications or hot spot mutations of *PIK3CA* can be found in various types of cancers as mentioned in Table 1. In prostate, breast, and melanoma, deletions, nonsense, and loss of function mutations of *PTEN* can be seen [19]. Gain-of-function missense mutations and amplifications of genes that encode three isoforms of AKT, which are also observed in human cancers. AKT1 E17K point mutation in the PH domain is the most frequent mutation [20]. Table 1 summarizes the frequency of mutations of *PIK3CA*, *AKT1*, and *PTEN* genes across various cancers.

### 3.1. PI3K Pathway in Hematological Malignancies

PI3KIs are heavily investigated in hematological malignancies. PI3K pathway activation occurs in hematological malignancies via the activation of receptor tyrosine kinase-mediated signaling such as B cell receptor (BCR). PI3Kδ isoform is selectively expressed by leukocytes and plays an important role in B-cell survival and migration while PI3Kγ is mostly expressed in T-cells and myeloid cells [26,27]. The reduction in cytokine production by T-cells can contribute to B-cell survival; thus, PI3Kδ and PI3Kγ isoforms are both targetable in hematological malignancies mostly in lymphomas [27]. There were four PI3KIs (copanlisib, idelalisib, duvelisib, and umbralisib) approved for hematological malignancies as further discussed below in the past decade; however, idelalisib has been withdrawn in FL and SLL, duvelisib in FL, and umbralisib in FL and marginal zone lymphoma (MZL).

### 3.2. PI3K Pathway in Breast Cancers

PI3KI alpelisib, AKT inhibitor capivasertib, and mTOR inhibitor everolimus are currently approved for hormone receptor-positive (HR) and human epidermal growth factor receptor 2-negative (HER2) breast cancer patients previously treated with endocrine therapy as further discussed below. *PIK3CA* mutation is shown to be a predictive marker for the PI3Kα inhibitor treatment [28]. The PI3K pathway is important in endocrine therapy-resistant breast cancers. The phosphorylation of estrogen receptor alpha (ERα) by PI3K and AKT activates ERα independently in the absence of estrogen. The upregulation of the PI3K pathway can lead to the loss of sensitivity of breast cancer cells to estrogen therapy [29]. *PIK3CA* mutations could be detected in up to 50% of ER-positive breast cancer patients [30]. This finding paved the way for the utilization of PI3K pathway inhibitors in patients with HR-positive breast cancers who progressed on endocrine therapy and their combination with endocrine therapy.

In patients with HER2 therapy-resistant breast cancer, increased HER3 expression by trastuzumab and *PIK3CA* mutation or PTEN loss can lead to PI3K activation. Thus, PI3KIs were investigated in combination with anti-HER2 therapy [31]. PI3K pathway activation can also cause chemotherapy resistance in breast cancer cells by increasing the outflow of chemotherapeutic drugs through adenosine triphosphate (ATP)-binding cassette transporter and the association between AKT phosphorylation and increased cell migration and apoptosis [32,33]. This led to the thought that targeting the PI3K pathway could restore the sensitivity of breast cancer cells to chemotherapy drugs, leading to PEGGY and BELLE-4 trials as discussed in detail below [34,35].

PI3K pathway activation is also involved in both primary and acquired resistance of breast cancer cells to CDK4/6 inhibitors. PDK1, one of the PI3K pathway components required for AKT activation, is a key modifier of ribociclib sensitivity [36]. The combination of CDK4/6 inhibitors with mTORC1/2 inhibitors has been demonstrated to cause breast cancer cell apoptosis and inhibit Rb (retinoblastoma) phosphorylation and E2F-mediated transcription in CDK4/6 resistant cells [37]. This led to the phase II BYLieve trial evaluating the combination of fulvestrant with PI3KI alpelisib after progression on a CDK4/6 inhibitor plus an aromatase inhibitor (AI) [38].

### 3.3. PI3K Pathway Alterations in Other Solid Tumors

PI3K pathway inhibitors have been heavily investigated in other solid tumors; however, there have been no FDA-approved PI3KIs to date. *PIK3CA* and *AKT* alterations have been found in 20–26% and 6% of advanced urothelial cancers, respectively [39]. Inactivation or deletion of *PTEN* was found in 13% of cases [39]. The clinical studies of PI3K pathway inhibitors in advanced urothelial cancers so far have not been promising, including buparlisib, sapanisertib, dactolisib, everolimus, and temsirolimus [40,41,42,43,44]. Given the cross-talk between the PI3K and the mitogen-activated protein kinase (MAPK) pathway, there is redundancy in the activation of downstream substrates between the two pathways. Therefore, the combination of BRAF and PI3KIs is one option to overcome redundancy pathways.

PI3K pathway plays an essential role in both endometrial and ovarian cancer development and progression. Alterations in PI3K pathways are present in more than 80% of endometrial cancers [45]. mTOR inhibitor temsirolimus has been evaluated as a single agent in heavily pretreated patients with advanced or recurrent endometrial cancer with 9% partial responses, whereas pilaralisib showed 6% ORR in patients with advanced or recurrent endometrial cancer with up to two prior lines of treatment [46,47]. In ovarian cancers, *PIK3CA* mutations seem to be the primary somatic mutations, comprising approximately 12% missense mutations in epithelial ovarian cancers while *PIK3R1* mutations are found in 3.8% of ovarian cancers [48,49]. Gain-of-function *PIK3CA* mutations are noted in 30–40% of clear-cell ovarian cancers [50]. The combination of everolimus and letrozole was shown to have 28% ORR in advanced or recurrent endometrial cancer [51]. The combination of mTOR inhibitor temsirolimus with chemotherapy paclitaxel and carboplatin demonstrated worse PFS compared to chemotherapy with bevacizumab [52]. In addition, the combination of everolimus and bevacizumab has lower ORR than the historical response with single-agent bevacizumab in patients with recurrent ovarian, peritoneal, and fallopian tube cancers [53,54].

The PI3K pathway plays an important role in the tumorigenesis and metastasis of colorectal cancer cells by affecting the metabolism and uptake of glucose and amino acids and participating in the cellular process [55]. mTOR signaling participates in vascular epidermal growth factor (VEGF)-mediated angiogenesis and AKT activates endothelial nitric oxide synthetase causing endothelial cell migration and angiogenesis [56]. However, clinical studies evaluating PI3K pathway inhibitors in colorectal cancers so far have not shown to have strong efficacy [57,58]. *PIK3CA* mutations are seen in 4–16.9% of gallbladder cancers and 0–9% of biliary tract cancers (BTC) while the positive immunostaining of AKT and increased mTOR gene copy number have been found in 46–100% and 48–92% of BTC, respectively [59]. However, despite strong preclinical evidence of PI3KIs in BTC, early-phase clinical trials of PI3K pathway inhibitors so far including buparlisib have not shown clinical benefit [59,60,61,62].

In head and neck cancers, *PIK3CA* mutations are one of the most frequent mutations in both HPV-positive and -negative head and neck squamous cell carcinoma (HNSCC) with *PIK3CA* mutations seen in 21% of HNSCC samples as per the Cancer Genome Atlas program (TCGA) [63]. *PTEN* loss has been reported in 2–24% of HNSCC cases [64,65]. Persistent AKT activation is also known to play a role in the mechanisms of resistance to cetuximab, thus indicating that the combination of PI3KI buparlisib and cetuximab could overcome cetuximab resistance [66,67]. On the other hand, the PI3K pathway is upregulated in 30–50% of prostate cancer patients [68]. The overexpression of AKT or suppression of PTEN can result in PI3K activation in prostate cancers. However, both PI3KIs and mTOR inhibitors have not shown to have promising clinical efficacy in clinical studies. AKT inhibitors might have some promising evidence as in the IPATential150 trial as discussed below but have intolerable adverse effects [69]. In thyroid cancers, BRAF and MEK inhibitors have been shown to increase PI3K pathway activation causing resistance to MAPK pathway inhibitors [70,71]. In melanomas, the loss of a functional *PTEN* gene leading to constitutive activation of PI3K signal transduction is observed in 10–35% percent of cases, causing resistance to BRAF inhibitors [72,73].

In a large study evaluating the next-generation sequencing (NGS) of tumor tissues of 1144 non-small cell lung cancer (NSCLC) patients, *PIK3CA* mutations were found in 3.7% of patients, with the highest mutations in squamous cell carcinoma (8.9%) and less common in adenocarcinoma (2.9%) [74]. The most common *PIK3CA* mutations were E545K exon 9 (57.1%), H1047R exon 20 (16.7%), and E542K exon 9 (14.3%) mutations. A total of 57.1% of those patients had coexisting oncogenic driver mutations including *EGFR*, *BRAF*, *ALK*, and *KRAS* mutations [74]. The upregulation of the mTOR and AKT was also demonstrated in NSCLC with up to 90% of phosphorylated mTOR (p-mTOR) in 90% of patients with adenocarcinoma, 60% of patients with large cell carcinoma, and 40% of patients with squamous cell carcinoma [75,76]. In total, 51% of 110 NSCLC tumor samples were found to have increased AKT activation in a study by Balsara et al. [77]. However, the clinical trials of PI3KIs including buparlisib in BASALT-1 trial and sonolisib have not been shown to have good clinical efficacy in NSCLC [78].

## 4. PI3K Inhibitors

Agents targeting class I PI3Ks have been explored for the treatment of various malignancies [11]. Class I PI3KIs are categorized into pan-PI3KIs, isoform-specific PI3KIs, and dual PI3K/mTOR inhibitors depending on their pharmacokinetic characteristics and their capacity to interact with ATP-binding clefts [5]. Initially, pan-PI3KIs were predominantly investigated but they were limited by various adverse effects and hematological targets. Therefore, iso-form selective PI3KIs were developed to overcome these limitations [79].

### 4.1. Pan-PI3K Inhibitors

Pan-PI3KIs are the first generation of PI3KIs that target all or most of the four catalytic isoforms of class I PI3Ks (α, β, δ, and γ) and include copanlisib, pictilisib, buparlisib, pilaralisib, and sonolisib [5,80]. Despite their widespread activities and successful early-phase clinical trials, further investigations of most pan-PI3KIs have been halted due to their adverse pharmacological events caused by both on-target and off-target effects including hyperglycemia, rash, diarrhea, colitis, hepatotoxicity, and hypertension [15].

*Copanlisib*, a potent pan-class I PI3KI with more specific activity against α and δ isoforms, was approved by the FDA on 14 September 2017 for adults with relapsed FL who have received at least two prior systemic therapies based on the results of the phase II single-arm CHRONOS-1 trial [81,82]. Copanlisib demonstrated promising efficacy in heavily pretreated patients with various subtypes of indolent and aggressive malignant lymphomas with an objective response rate (ORR) of 58.7% and a median duration of response (DOR) of 12.2 months. The most frequent adverse events are hyperglycemia, hypertension, diarrhea, fatigue, neutropenia, and thrombocytopenia. Treatment-related adverse effects leading to the discontinuation of the drug were seen in 16% of patients [83] (Refer to Table 2).

The phase III CHRONOS-3 trial showed that copanlisib can be safely and effectively combined with rituximab in relapsed indolent non-Hodgkin’s Lymphoma (NHL) with improved progression-free survival (PFS) of 21.5 months in copanlisib and rituximab arm vs. 13.8 months in a single rituximab arm (HR (hazard ratio) 0.52, 95% CI 0.39–0.69; *p* < 0.0001) [90]. Its combination with gemcitabine and cisplatin in advanced and unresectable BTC as first-line treatment did not meet its primary endpoint PFS at 6 months. It showed a median overall survival (OS) of 13.7 months, and a median PFS of 6.2 months, with the most common adverse events being decreased neutrophil count, anemia, increased lipase level, and hypertension [91]. Phase I trial of copanlisib in combination with cetuximab in heavily pretreated patients with recurrent and/or metastatic HNSCC was stopped early due to unfavorable toxicities, especially hyperglycemia, and limited efficacy [92]. In the subprotocol Z1F of phase II NCI-MATCH ECOG-ACRIN trial (EAY131), copanlisib demonstrated ORR of 16% (90% CI 6–33; *p* = 0.0341 against null rate of 5%) in 20 different advanced pretreated solid tumors with *PIK3CA* mutations, meeting its primary endpoint and showing potential clinical benefit in select patients with PI3KCA mutations in refractory settings [93]. However, its application was withdrawn from the FDA on 13 November 2023, after its evaluation in the confirmatory phase III CHRONOS-4 trial of the addition of conpalisib to standard rituximab-based immunochemotherapy did not meet its primary endpoint PFS [94,95].

*Pictilisib (GDC-0941)* is an ATP competitive oral pan-class I PI3KI and the first PI3KI to be assessed in a clinical trial. Pictilisib was investigated in combination with fulvestrant in a phase II FERGI trial with HR-positive, HER2-negative, and AI-resistant advanced breast cancer. It showed no significant improvement in PFS but caused serious toxicities including rash, diarrhea, fatigue, and transaminitis leading to dose limitations and more discontinuations of pictilisib than the placebo-fulvestrant group [96]. In a phase II PEGGY trial, pictilisib in combination with paclitaxel in HR-positive, HER2-negative locally recurrent or metastatic breast cancer showed no added benefit to paclitaxel alone [34]. Pictilisib in combination with paclitaxel with or without bevacizumab, trastuzumab, or letrozole has shown potential antitumor activity with ORR in 33% of patients with locally recurrent or metastatic breast cancer [97]. However, pictilisib in combination with MEK inhibitor cobimetinib, or EGFR inhibitor erlotinib in patients with advanced solid tumors in phase I studies showed limited efficacy [98,99]. Piclitaxib is currently being evaluated in a phase II trial of previously untreated CD30-negative peripheral T-cell lymphomas (NCT04803201).

*Buparlisib (BKM120)* is another ATP competitive oral pan-class I PI3KI, which has been investigated in several trials including breast, renal cell, lung, glioblastomas, and head and neck cancers with unfavorable safety profile and minimal antitumor activity in some trials [100,101,102]. It was evaluated in the phase II BASALT-1 trial in patients with relapsed NSCLC with *PIK3CA* or *PTEN* alterations, but only two patients had partial responses and the second stage of the study did not proceed given its futility [78]. On the other hand, buparlisib in combination with paclitaxel in patients with platinum-pretreated recurrent or metastatic HNSCC in phase II randomized BERIL-1 trial demonstrated significantly longer PFS in the buparlisib group [103]. PFS was 4.6 months in the buparlisib group vs. 3.5 months in the placebo group (HR 0.65, 95% CI 0.45–0.95, nominal one-sided *p* = 0.011), but serious adverse effects were observed more in the buparlisib group (57% vs. 47%) [103]. The most common grade 3 or more adverse events were hyperglycemia, anemia, neutropenia, and fatigue [103]. Buparlisib in combination with imatinib showed no added benefit in previously treated gastrointestinal stroma tumors nor in combination with enzalutamide in previously treated men with metastatic castration-resistant prostate cancer (mCRPC) [104,105].

Buparlisib was heavily investigated in breast cancers with the thought that targeting both PI3K and estrogen receptors can prevent estrogen receptor (ER) activation in endocrine therapy-resistant tumors and overcome mTOR feedback activation [106,107]. In two phase III randomized placebo-controlled BELLE-2 and BELLE-3 trials, the combination of buparlisib with fulvestrant was evaluated in HR-positive, HER2-negative postmenopausal breast cancer patients who progressed on AI or mTOR inhibitors. The buparlisib group had a PFS benefit of 2.8 months and 2.1 months compared to the placebo group in BELLE-2 and BELLE-3 trials, respectively [106,108]. Both trials met the primary endpoint PFS with the addition of buparlisib but more serious adverse events including transaminitis, hyperglycemia, and rash were reported in the buparlisib group, leading to more discontinuations in the buparlisib group [106,108]. Subgroup analysis in both trials confirmed that the presence of *PIK3CA* mutations was associated with improvement in PFS. The safety profile is not desirable but the efficacy of buparlisib supports the rationale for the use of PI3K inhibitors plus endocrine therapy in breast cancer patients with *PIK3CA* mutations. Its combination with tamoxifen in the phase II PIKTAM trial in HR-positive, HER2-negative advanced breast cancer patients was terminated early due to its risk-benefit profile despite its promising efficacy in the *PIK3CA*-mutated group with a median PFS of 8.7 months, ORR of 40%, and DCR of 80% [109].

However, the addition of buparlisib to paclitaxel in HR-positive, HER2-negative advanced breast cancer patients in the phase II/III BELLE-4 trial did not improve PFS in all or PI3K pathway-activated study populations, and the trial was stopped for futility at the end of phase II [35]. Buparlisib with trastuzumab demonstrated limited efficacy in patients with heavily pretreated trastuzumab-resistant HER2-positive breast cancer, whereas the phase II NeoPHOEBE trial showed that the combination of neoadjuvant buparlisib plus trastuzumab and paclitaxel for HER2-positive primary breast cancer was not feasible due to significant liver toxicity [110,111].

Buparlisib can cross the blood–brain barrier; however, it has not demonstrated sufficient anti-tumor activity in combination with carboplatin and lomustine in a phase Ib/II trial in patients with recurrent glioblastoma previously treated with radiotherapy and temozolomide or as a single-agent in patients with PI3K-activated recurrent glioblastoma [112,113]. Moreover, given its PI3K inhibition in the central nervous system (CNS), it could cause CNS toxicity and lead to mood alterations including anxiety, hallucinations, and irritability [114]. Currently, the phase III BURAN trial (NCT04338399) is evaluating the combination of buparlisib and paclitaxel in patients with advanced HNSCC who progressed on immunotherapy alone or in combination with a platinum-based regimen [115].

*Pilaralisib (SAR245408, XL147)* is another pan-class I PI3KI, which has shown a favorable safety profile but limited activity in some trials. It demonstrated a minimal antitumor activity (ORR 6%) in patients with advanced or recurrent endometrial carcinoma who progressed after first-line chemotherapy and in combination with letrozole in patients with HR-positive, HER2-negative metastatic breast cancer (ORR of 4%) [47,116]. The most common adverse effects were rash, diarrhea, and fatigue [47,116].

*Sonolisib (PX-866)* is another pan-class I PI3KI that has been investigated in several phase II trials. Sonolisib in combination with docetaxel did not improve PFS, ORR, or OS in phase II trials of patients with refractory advanced NSCLC and HNSCC without molecular preselection, in combination with cetuximab in metastatic colorectal cancer (mCRC), or in recurrent glioblastomas [58,117,118,119].

### 4.2. Isoform-Specific PI3K Inhibitors

Compared to pan-PI3KI, isoform-specific PI3KIs have a better therapeutic window and safety profile given a high potency toward specific PI3K isoform [120]. Isoform-specific PIKIs have been evaluated in several clinical trials and a few of those had FDA approvals.

#### 4.2.1. PI3Kα Inhibitors

*Alpelisib* is the only selective PI3Kα inhibitor and the first PI3KI approved by the FDA on 24 May 2019 for the treatment of HR-positive, HER2-negative, *PIK3CA*-mutated, advanced or metastatic breast cancer in combination with fulvestrant in postmenopausal female and male patients previously treated with endocrine therapy based on SOLAR-1 phase III trial [84,121]. Patients were divided into a cohort with *PIK3CA* mutation and a cohort without *PIK3CA* mutation. In each cohort, patients were randomized to the alpelisib-fulvestrant group and the placebo-fulvestrant group. Combined alpelisib and fulvestrant showed better median PFS of 11 months compared to 5.7 months in the placebo-fulvestrant group (HR 0.65, 95% CI 0.5–0.85; *p* = 0.001) in patients with *PIK3CA*-mutated cancer [84] (refer to Table 2). However, it had 25% discontinuation rates compared to 4.2% in the placebo group with grade 3 or 4 hyperglycemia, rash, and diarrhea [84]. In addition, when SOLAR-1 was designed, CDK4/6 inhibitors were not yet approved as a standard treatment for advanced HR-positive, HER2-negative breast cancer. Therefore, the phase II BYLieve trial evaluated patients with *PIK3CA*-mutated, HR-positive, HER2-negative advanced breast cancer after progression on a CDK4/6 inhibitor plus an AI and alpelisib continued to show efficacy in combination with fulvestrant [38]. However, alpelisib was shown to be not effective as a neoadjuvant treatment in combination with letrozole in early-stage HR-positive, HER2-negative breast cancer in NEO-ORB phase II trial [122]. Currently, alpelisib is being evaluated in combination with fulvestrant in patients with HR-positive, HER2-negative advanced breast cancer after treatment with CDK4/6 inhibitors and AI in phase III EPIK-B5 trial (NCT05038735). It is also being investigated in combination with trastuzumab with or without fulvestrant compared to trastuzumab plus chemotherapy in previously treated patients with *PIK3CA*-mutated HER2-positive advanced breast cancer in phase III ALPHABET trial (NCT05063786) [123,124].

*Taselisib (GDC-0032)* is a predominant PI3Kα isoform-specific inhibitor that has been extensively investigated in phase I, II, and III trials [16]. Taselisib plus letrozole have shown clinical benefit as a neoadjuvant treatment in postmenopausal women with ER-positive, HER2 negative early-stage I–III resectable breast cancer in randomized phase II LORELEI trial with better ORR of 50% in the taselisib-letrozole group vs. 39% in the placebo-letrozole group (odds ratio 1.55, 95% CI 1.0–2.38; *p* = 0.033) with improved ORR of 56% among *PIK3CA*-mutated patients [125]. The most common adverse events were gastrointestinal issues, infections, and skin disorders [125]. On the other hand, taselisib was evaluated in previously treated *PIK3*-mutant patients with stage IV squamous cell lung cancer in the Lung-MAP trial, but the trial did not meet its primary endpoint response rate and was closed after interim futility analysis [126]. Moreover, another phase II study of taselisib in heavily treated PIK3CA-mutated solid tumors in NCI-MATCH ECOG-ACRIN trial (EAY 131) subprotocol I showed no complete or partial responses with the best response of stable disease in 32 out of 61 patients (52%) [127]. In the phase III SANDPIPER trial of taselisib in combination with fulvestrant in HR-positive, HER2-negative, advanced breast cancer patients pretreated with AI also demonstrated modest clinic benefit with only 2 month PFS advantage in the taselisib arm (7.4 vs. 5.4 months, HR 0.7, 95% CI 0.56–0.89; *p* = 0.0037). However, it caused significant serious adverse events (32% vs. 8.9%) leading to a higher rate of discontinuations (16.8% vs. 2.3%) and dose reductions (36.5% vs. 2.3%) compared to the placebo-fulvestrant group [128]. The most common serious adverse events were diarrhea, hyperglycemia, nausea, decreased appetite, and stomatitis [128]. Based on the results of the SANDPIPER trial, there was no further development of taselislib by its pharmaceutical company.

*Inavolisib (GDC-0077)* is another PI3Kα specific inhibitor that also degrades mutant p110α [129,130]. It was evaluated in the phase I trial as a single agent or in combination with palbociclib with or without letrozole or fulvestrant in *PIK3CA*-mutant HR-positive, HER2-negative solid tumors and showed acceptable long-term tolerability [129]. The most common all-grade adverse events were hyperglycemia, stomatitis, neutropenia, and diarrhea with neutropenia and hyperglycemia being the most common grade 3 or higher adverse effects [129]. It is currently being investigated in several trials including phase III trial of *PIK3CA*-mutated HER2-positive advanced breast cancer patients in combination with anti-HER2 and endocrine therapy (NCT05894239). It is also being investigated in combination with palbociclib and fulvestrant in *PIK3CA*-mutated HR-positive, HER2-negative metastatic breast cancer in INAVO120 trial (NCT04191499) as well as in combination with fulvestrant compared to alpelisib plus fulvestrant in HR-positive, HER2-negative *PIK3CA*-mutated advanced breast cancers (NCT05646862).

*Risovalisib (CYH-33)* is a highly selective PI3Kα inhibitor [131]. It has shown synergic activity with CDK4/6 inhibitors in KRAS-mutated NSCLC cell line models, as well as with radiation in esophageal squamous cell cancer cell lines in preclinical studies [131,132]. In addition, risovalisib demonstrated a synergistic effect with KRASG12C inhibitor AMG510 (sotorasib) against parental and resistant KRASG12C-mutant cells in both in vitro and in vivo. This concomitant inhibition of PI3K and MAPK pathway signaling provides a rationale for the potential combination of PI3Kα inhibitors with KRASG12C inhibitors in KRASG12C-mutant cancers [133]. Its first-in-human phase Ia trial in pretreated solid tumors with *PIK3CA* mutations showed its tolerability and efficacy in several types of *PIK3CA*-mutant solid tumors such as breast, ovarian, and gastric cancers [134]. The most common grade 3 or higher adverse events were hyperglycemia, rash, thrombocytopenia, edema, and fatigue [134]. It is currently investigated in phase I and II trials in advanced solid cancers including ovarian, prostate, endometrial, and breast cancers.

*Serabelisib (TAK117)* is another PI3Kα inhibitor that was evaluated in the early phase 1 trial in combination with mTORC 1/2 inhibitor sapanisertib and paclitaxel in advanced ovarian, endometrial and breast cancer patients. It demonstrated a tolerable safety profile with an ORR of 46% [135]. However, its combination with sapanisertib in previously treated patients with advanced renal cell cancer (RCC) who progressed on VEGF-targeted therapies did not improve efficacy compared to everolimus [136]. It is currently being evaluated in combination with nab-paclitaxel in advanced solid tumor patients with *PI3CA* mutation and *PTEN* loss of function mutation (NCT05300048).

#### 4.2.2. PI3Kβ Inhibitors

First-in-human phase 1 trials of PI3Kβ inhibitors, SAR260301, and GSK2636771 are available, but the clinical investigation of SAR260301 was not further pursued due to its rapid clearance making it insufficient to inhibit the PI3K pathway [137,138].

*GSK2636771* is a selective ATP competitive, oral PI3Kβ inhibitor. It was evaluated in a phase I trial of patients with *PTEN*-deficient mCRPC who progressed on prior enzalutamide with the hope of overcoming resistance to androgen deprivation therapy. It demonstrated an acceptable safety profile but with limited anti-tumor activity [139]. PTEN is a negative regulator of the PI3K pathway and the loss of PTEN protein is seen in 40–60% of mCRPC [139]. Its combination with paclitaxel also demonstrated antitumor activity with an ORR of 17.9% and DCR of 67.9% in patients with PTEN-deficient advanced gastric cancer who progressed from first-line therapy (fluoropyrimidine and platinum) in a phase Ib/II trial [140]. It appears that the complete loss of PTEN expression may be associated with the clinical benefit from GSK2636771 and paclitaxel, which warranted further analysis. The antitumor activity of GSK2636771 is being further evaluated as a single agent in the NCI-MATCH clinical trials and in combination with pembrolizumab in melanoma with PTEN loss (NCT03131908).

*AZD8186* is a selective dual PI3Kβ/δ inhibitor that has completed phase I trial in previously treated patients with advanced solid tumors with *PTEN* deficiency/mutation or *PIK3CB* mutation. It demonstrated limited efficacy with only one partial response in a patient with *PTEN*-deficient mCRC [57]. Its combination with paclitaxel in patients with *PTEN*-deficient or *PIK3CB*-mutated advanced gastric cancer showed tolerability but again with limited efficacy of 18.8% partial response and DOR of only 1.8 months [141].

#### 4.2.3. PI3Kδ Inhibitors

Given that PI3Kδ isoform is highly expressed in lymphoid tissues and takes part in B cell receptor signaling, B cell proliferation, and survival, PI3Kδ inhibitors are widely explored in lymphoid disorders [85].

*Idelalisib* is an oral ATP competitive PI3Kδ inhibitor which was the first PI3KI that received FDA approval on 23 July 2014 for the treatment of indolent B-cell malignancies, including relapsed/refractory (R/R) CLL, in combination with rituximab, as well as monotherapy for relapsed FL and SLL, in patients who received at least two prior systemic therapies [9]. The approval was based on a phase III randomized trial of idelalisib in combination with rituximab vs. rituximab-placebo in patients with relapsed CLL [85]. Primary endpoint PFS was not reached in the idelalisib-rituximab group vs. 5.5 months in the rituximab-placebo group (HR 0.15; *p* < 0.001) with better ORR of 81% compared to 13% (odds ratio 29.92; *p* < 0.001) [85]. It continued to show an improved ORR of 85.5%, PFS of 20.3 months, and median OS of 40.6 months in extended follow-up studies without new idelalisib-related adverse events [142]. In a single-arm phase II trial, it demonstrated antitumor activity with ORR of 57% including a 6% complete response, PFS of 11 months, and OS of 20.3 months in heavily treated patients with indolent NHL with prior rituximab and alkylating agents [86]. The most common adverse events were neutropenia, transaminitis, diarrhea, and pneumonia [86]. However, it only showed modest activity with an ORR of 20%, median DOR of 8.4 months, and median PFS of 2.3 months in the phase II trial of patients with R/R classical Hodgkin lymphoma (cHL) [143]. Idelalisib plus ofatumumab combination demonstrated better PFS (16.3 months vs. 8.0 months in the ofatumumab alone group) in the phase III trial of patients with relapsed CLL, including in those with high-risk disease [144]. However, idelalisib was voluntarily withdrawn from its approval for FL and SLL given its difficulty in enrolling patients for the confirmatory trial [145]. It is currently being evaluated in previously metastatic or recurrent NSCLC (NCT03257722) and previously treated CLL/SLL (NCT04666038).

*Duvelisib (INK 1197, IPI-145)* is a PI3Kδ/γ inhibitor that was FDA-approved on 24 September 2018 for the treatment of R/R CLL/SLL and FL after at least two prior therapies, based on the results of phase III DUO trial [87,146]. However, its indication for FL was withdrawn by its parent company to emphasize its development on T cell lymphomas, and the FDA has withdrawn its approval for use in CLL/SLL due to unfavorable risk and benefit profile in September 2022 [147,148]. Phase III randomized DUO trial evaluated duvelisib vs. ofatumumab in patients with R/R CLL/SLL and demonstrated that duvelisib has a PFS benefit of 3.4 months (HR 0.52; *p* < 0.0001) including high-risk disease with del(17p) and/or TP53 mutations, and ORR was 74% higher in the duvelisib group compared to 45% in the ofatumumab group (*p* < 0.0001) [87]. The most common adverse effects of duvelisib were diarrhea, neutropenia, pyrexia, nausea, anemia, and cough while the most common grade 3 or higher adverse effects were neutropenia, diarrhea, pneumonia, and anemia [87]. Davids et al. reported that duvelisib demonstrated a high ORR of 77% with a DOR of 14.9 months and PFS of 15.7 months in patients with R/R CLL/SLL who progressed on ofatumumab based on a crossover of patients who received ofatumumab in DUO trial [149]. In the phase II DYNAMO trial, duvelisib also demonstrated clinically meaningful efficacy with an ORR of 47.3% (SLL, 67.9%; FL, 42.2%; MZL 38.9%), with a DOR of 10 months and a PFS of 9.5 months in heavily pretreated indolent NHL refractory to rituximab and chemotherapy or radioimmunotherapy, providing a potential oral treatment option for the elderly [88]. Its phase I trial in combination with rituximab or rituximab and bendamustine in NHL or CLL showed a tolerable safety profile with ORR of 71.8%, median PFS of 13.7 months, and 30-month OS of 62% [150]. However, its investigations in phase III trials including DYNAMO + R comparing duvelisib and rituximab to rituximab alone in patients with FL (NCT02204982) and BRAVURA comparing duvelisib, rituximab and bendamustine with rituximab and bendamustine in patients with NHL (NCT02576275) were withdrawn by the sponsor as they were not expected to lead to FDA approval [151]. Duvelisib is currently being investigated in several trials including unresectable melanomas, recurrent or metastatic HNSCC, and hematological malignancies including T and NK cell lymphomas.

*Tenalisib(RP6530)* is another PI3Kδ/γ inhibitor. Its evaluation in the first-in-human phase I trial of patients with R/R hematologic malignancies demonstrated an ORR of 19% and a DOR of 5.7 months with asthenia, cough, pyrexia, and gastrointestinal disorders being the most frequent adverse events [152]. It was then investigated in an early phase I/Ib trial as a single agent in R/R peripheral and cutaneous T cell lymphoma (TCL) with an ORR of 45.7% and a DOR of 4.9 months with transaminase elevations being the most frequent grade 3 or higher adverse event [153]. The combination of tenalisib and romidepsin in patients with R/R TCL demonstrated promising clinical activity with an ORR of 63% (CR 25.9% and PR 37%) and a median DOR of 5.03 months [154]. The combination of tenalisib and romidepsin has shown complete response in a patient with R/R Sezary syndrome and a long duration of response was noted with tenalisib monotherapy [155].

*Umbralisib (TGR-1202, Rp-5264)* is a dual inhibitor of PI3Kδ and casein kinase-1e (CK1e), which was FDA-approved on 5 February 2021 for the treatment of R/R MZL after at least one prior anti-CD20-based regimen, and the treatment of R/R FL after at least three prior lines of systemic therapy [156]. The approval was based on the phase II UNITY-NHL trial of umbralisib in heavily pretreated patients with indolent NHL (MZL, FL, and SLL), which showed an ORR of 47.1% in the overall population (MZL 49.3%, FL 45.3%, and SLL 50.0%) with the most common adverse effects being diarrhea, nausea, fatigue, vomiting, and cough [89]. However, in the phase III UNITY-CLL trial, umbralisib in combination with anti-CD20 monoclonal antibody ublituximab was compared to obinutuzumab and chlorambucil in treatment-naive and R/R CLL. It initially showed improved PFS but later demonstrated safety concerns and inferior OS [157,158]. Phase II trial investigating the addition of umbralisib to JAK1/2 (Janus Kinase 1/2) inhibitor ruxolitinib in patients with myelofibrosis (MF) demonstrated that combination treatment was well tolerated with an ORR of 37% and that umbralisib may re-sensitize patients with MF to JAK1/2 inhibitor ruxolitinib [159]. However, the FDA withdrew its approval on 1 June 2022, due to increased serious adverse events and risk of death with the use of umbralisib with updated findings from the UNITY-CLL trial [158].

*Parsaclisib (INCB050465)* is a highly selective, next-generation PI3Kδ inhibitor. It demonstrated efficacy with an ORR of 25.5% and a median DOR of 6.2 months in patients with R/R diffuse large B cell lymphoma (DLBCL) in the phase II CITADEL-202 trial [160]. Adverse events included rash, colitis/diarrhea, transaminitis, and pancytopenia with the most serious adverse effects being abdominal pain, hyperglycemia, and pyrexia [160]. It demonstrated antitumor activity with potential for long-term improved outcomes in R/R B-cell malignancies with an ORR of 71% in FL, 78% in MZL, 67% in mantle cell lymphoma (MCL), and 30% in DLBCL in another phase I/II study of R/R B cell malignancies [161]. It is currently being investigated in multiple hematological malignancies.

*Linperlisib (YY20394)* is a novel orally active PI3Kδ inhibitor that has demonstrated clinical activity with an ORR of 79.8%, a median DOR of 12.3 months, and a median PFS of 13.4 months for patients with R/R FL in a single-arm phase II trial who had received at least two prior systemic therapies [162]. The most frequent grade 3 or higher adverse events were infectious pneumonia, neutropenia, leukopenia, increased lipase level, thrombocytopenia, hypertriglyceridemia, and interstitial lung disease [162]. Linperlisib has promising efficacy with deep and durable responses demonstrating ORR of 60% and median DOR of 15 months in R/R peripheral TCL patients with tolerable toxicity [163]. It is currently being investigated in the phase I/II trials of TCL and lymphomas.

*AMG319* is another highly selective PI3Kδ inhibitor that was evaluated in the first-in-human trial in patients with R/R CLL and NHL with efficacy observed in CLL patients with high-risk cytogenetics [164]. The most common grade 3 or higher adverse effects were colitis, anemia, leukocytosis, infection, and hemolysis [164].

#### 4.2.4. PI3Kγ Inhibitors

*Eganelisib (IPI 549)* is a first-in-class, selective oral PI3Kγ inhibitor that has shown antitumor activity alone or in combination with programmed cell death protein 1/ligand 1 (PD-1/PD-L1) inhibitors in preclinical studies [165]. PI3K inhibition can cause a reduction in the number of regulating T (T reg) cells, intra-tumoral infiltration of CD4+ and CD8+ T cells, and the production of immunostimulatory cytokines in the tumor microenvironment, thus stimulating anticancer immunity [166]. Eganelisib in combination with nivolumab compared to nivolumab monotherapy in phase II MARIO-275 trial of advanced urothelial carcinoma patients with at least 1 prior line of platinum-based chemotherapy regimen has demonstrated improved ORR of 30.3% and PFS of 9.1 months in the eganelisib arm compared to ORR of 25% and PFS of 8.0 months in the nivolumab alone arm during preliminary data reports [165]. Safety run-in report of eganelisib plus atezolizumab and nab-paclitaxel as a first-line treatment for locally advanced or metastatic triple-negative breast cancer patients in phase II MARIO-3 trial demonstrated manageable toxicity and promising anti-tumor activity with ORR of 100% irrespective of biomarker status and the results of the expansion study are currently pending [167]. The most common adverse events were neutropenia, fatigue, diarrhea, hyperglycemia, transaminase elevation, pyrexia, and rash [167]. Its phase I MARIO-1 trial showed anti-tumor activity in patients with advanced solid tumors who progressed on prior lines including PD1/PD-L1 inhibitors with tolerable safety profile either as a monotherapy or in combination with nivolumab [168]. It is currently evaluated in phase I/II trials of advanced solid tumors including HNSCC.

*Zandelisib (ME-401)* is another PI3Kγ inhibitor that initially received FDA fast-track designation for the treatment of patients with R/R FL who have received at least 2 prior systemic therapies on 31 March 2020 [169]. Zandelisib has completed first-in-human, dose-escalation, and dose-expansion phase 1 trials in R/R B cell malignancy as a monotherapy or in combination with rituximab [170]. On the other hand, the combination of zandelisib plus zanubrutinib was well tolerated in the phase II trial of patients with R/R FL or MCL with high response rates in R/R FL (ORR of 80%) and MCL (ORR of 76%), and the responses seemed to deepen over time [171]. Its phase II TIDAL trial in R/R FL and phase III COSTAL trial comparing its combination with rituximab to rituximab and chemotherapy in relapsed indolent NHL and CLL have been terminated despite its promising ORR of 70.3% in the TIDAL trial due to the discontinuation of zandelisib [151,172,173].

#### 4.2.5. Dual PI3K/mTOR Inhibitors

There has been an emerging interest in the development of agents that inhibit both PIK3CA and mTOR, thus achieving a more complete blockade by inhibiting multiple points in the PI3K/AKT/mTOR pathway and omitting a negative feedback loop [174]. Dual inhibitors of PIK3CA and mTOR target the active sites of both holoenzymes, inhibiting the pathway both upstream and downstream of AKT, thus avoiding the problem of paradoxical AKT activation, which is known to occur with mTORC1 (mammalian target of rapamycin complex 1) blockers [174]. Therefore, they have been associated with higher anti-tumor activity but also with higher adverse events [175]. Dactolisib, voxtalisib, bimiralisib, and gedatolisib are some of the agents that have been evaluated in phase I/II trials [175]. To date, there are no dual PI3K/mTOR inhibitors approved for cancer treatment even though they are currently being investigated in clinical trials.

*Dactolisib (BEZ235)* is a dual PI3K/mTOR kinase inhibitor that has been investigated in preclinical, phase I, and II trials but has demonstrated poor efficacy and tolerability in advanced pancreatic neuroendocrine tumors, RCCs, prostate cancers, and advanced solid tumors [176,177,178,179]. The most common adverse events were diarrhea, stomatitis, and nausea [176].

*Apitolisib (GDC-0980, RG7422)* has limited anti-tumor activity due to poor tolerability, especially in diabetic patients at higher doses, multiple on-target adverse events, and has been investigated in various cancers including recurrent or persistent endometrial cancers, advanced solid tumors, mCRPC (in combination with abiraterone), and RCCs [180,181,182,183]. The most common grade 3 or 4 adverse events were hyperglycemia, rash, colitis, and pneumonitis [180].

*Gedatolisib (PKI-587)* is an intravenous, ATP-competitive, pan-PI3K/mTOR inhibitor that has demonstrated significant antitumor activity in preclinical models [184]. FDA has granted a fast-track designation to gedatolisib for use as a potential therapeutic option in patients with HR-positive, HER2-negative metastatic breast cancer who progressed on prior CDK4/6 therapy [185]. It has been evaluated in early phase I studies in patients with advanced solid tumors and triple-negative breast cancer and showed an acceptable tolerability profile and promising clinical activity with an ORR of 40% [107]. The most common adverse effects were anemia, nausea, and fatigue [107]. In another phase Ib expansion trial, gedatolisib in combination with palbociclib and endocrine therapy showed antitumor activity in women with HR-positive metastatic breast cancer with an ORR of 84% in the group used as a first-line treatment, an ORR of 75% as a second-line treatment, and an ORR of 33–64% in patients with prior CDK4/6 inhibitor therapy [186,187]. Currently, gedatolisib is being evaluated in the phase III VIKTORIA-1 trial combined with fulvestrant with or without the CDK4/6 inhibitor palbociclib in patients with HR-positive, HER2-negative advanced breast cancer who progressed on CDK 4/6 therapy (NCT05501886).

*Voxtalisib (SAR245409, XL 765)* is a reversible potent pan PI3K/mTOR inhibitor and has been studied in HR-positive metastatic breast cancer refractory to AI, R/R NHL, or CLL, with temozolomide in high-grade gliomas, and with pimasertib in advanced solid tumors; however, it has limited activity observed across multiple clinical trials [116,188,189,190,191]. The most frequent adverse effects were diarrhea, fatigue, nausea, dyspnea, and pyrexia [188,189].

*Bimiralisib*, another oral dual PI3K/mTOR inhibitor, demonstrates tolerability but some efficacy with an ORR of 17% in chemotherapy and radiotherapy-resistant patients with HNSCC and *NOTCH1* loss-of-function mutation in both phase I and II trials [192,193]. Bimiralisib has also been shown to have efficacy in combination with BCL2 (B-cell lymphoma 2) inhibitor venetoclax in acute myeloid leukemia (AML) with *IDH2, NPM1, and FLT3* mutations in preclinical studies [194].

*Samotolisib (LY3023414)* is an oral ATP-competitive PI3K/mTOR dual kinase and DNA-dependent protein kinase inhibitor which was evaluated in phase Ib/II trial in combination with enzalutamide in patients with mCRPC who progressed on abiraterone [195]. Samotolisib in combination with enzalutamide has shown an improved radiographic PFS benefit of 4.7 months (*p* = 0.03) in patients and better PFS in patients without androgen receptor splice variant 7 with tolerable side effects [195].

#### 4.2.6. Isoform and Mutant Selective PI3Kα Inhibitors

The search for improved PI3KI led to the development of allosteric mutant and isoform-selective PI3Kα inhibitors. Recurrent oncogenic *PIK3CA* mutations are observed far from the ATP binding sites of current PI3KI and the lack of an allosteric binding site on wild type target or other isoforms reduces the affinity of those drugs. Isoform and mutant selective PI3Kα inhibitors are strong inhibitors of only mutant proteins, thus sparing the wild-type PI3K and leading to the avoidance of dysregulation in glucose metabolism, which is one of the most dose-limiting toxicities of PI3KIs [120]. Currently, RLY-2608 and LOXO-783 from this class have been investigated in clinical trials. RLY-2609 inhibits helical domain (E542K and E545K) and kinase domain (H1047R) mutations with a 10-fold affinity compared with wild-type *PIK3CA* and more than 200-fold selectivity compared to other PI3K isoforms while LOXO-783 selectively inhibits kinase domain *PIK3CA* H1047R mutation with a 90-fold selectivity compared to wild type *PIK3CA* and no activity on other PI3K isoforms [120]. ST-814 is a CNS-penetrant, mutant selective allosteric PI3Kα H1047X inhibitor that has a 14-fold selective activity over PI3Kα H1047X compared to PI3Kα wild-type and has been shown to be comparable to alpelisib without significant hyperglycemia in preclinical study [196].

## 5. AKT/mTORC Inhibitors

### 5.1. AKT Inhibitors

AKT is another substrate in the PI3K pathway that can be targeted for the inhibition of the PI3K pathway. AKT inhibitors currently being evaluated in clinical trials are pan-AKT inhibitors such as capivasertib, afuresertib, and apatasertib. Among AKT inhibitors, only capivasertib has received FDA approval [197].

*Capivasertib* was the first of its kind to be approved by the FDA as a pan-AKT inhibitor (AKT 1/2) for adult patients with HR-positive, HER2-negative locally advanced or metastatic breast cancer patients with *PIK3CA/AKT1/PTEN*-alterations in August 2023 [197]. CAPItello-291 was a phase-3 trial that studied the effectiveness and safety of capivasertib vs. fulvestrant in HR-positive, HER2-negative breast cancer patients who had received AI treatment with or without CDK 4/6 inhibitors. The study’s primary endpoint was to assess the investigator-assessed PFS in the overall population and *PIK3CA*, *AKT1*, or *PTEN*-altered tumors and safety [198]. For the overall population of patients, the median PFS was higher in the capivasertib–fulvestrant arm (7.2 months) compared to the placebo-fulvestrant group (3.6 months) (HR 0.6, 95% CI 0.51–0.71; *p* < 0.001). In the *AKT*-altered population, patients had a median PFS of 7.3 months in the capivasertib–fulvestrant arm vs. 3.1 months in the placebo-fulvestrant arm (HR 0.50, 95% CI, 0.38 to 0.65; *p* < 0.001) [198]. In the capivasertib–fulvestrant group, the most common any-grade adverse events were diarrhea (72.4%), rash (38%), and nausea (34.6%), while hyperglycemia was only observed in 16.3% of patients. The most common grade 3 or higher adverse events were rash (12.1%) and diarrhea (9.3%). The discontinuation rate due to adverse events was 13% in the capivasertib group compared to 2.3% in the placebo–fulvestrant arm [198].

*Afuresertib (GSK2110183)* is an oral ATP competitive pan-AKT inhibitor that has shown a tolerable safety profile and some clinical efficacy in multiple myeloma, Langerhans cell histiocytosis, recurrent platinum-resistant ovarian cancer in combination with carboplatin and paclitaxel; however, its combination with ofatumumab did not show benefit over single-agent ofatumumab [199,200,201,202]. It has been shown to inhibit cell proliferation of Merkel cell carcinoma via the deactivation of mTOR and glycogen synthase kinase 3 pathway proteins and the activation of pro-apoptotic pathways in preclinical studies [203]. It has also demonstrated the inhibition of malignant pleural mesothelioma cells by G1 phase cell cycle arrest and increased apoptotic cells in preclinical studies [204].

*Ipatasertib (GDC0068)* is an ATP-competitive pan-AKT inhibitor, which has been shown to have some efficacy in combination with carboplatin in uterine serous carcinoma, in combination with erdafitinib in bladder cancer cells, as well as to increase the sensitivity of endometrial cancer cells to paclitaxel in preclinical studies [205,206,207]. It also demonstrated clinical efficacy in combination with abiraterone in patients with metastatic prostate cancer with or without PTEN loss, in combination with paclitaxel in locally advanced or metastatic triple-negative breast cancer in LOTUS trial, and in combination with abiraterone in mCRPC with PTEN loss in IPATential150 [208,209,210]. However, it did not show clinical efficacy in combination with mFOLFOX (modified fluorouracil and oxaliplatin) in locally advanced or metastatic gastric or gastroesophageal junction cancer, or in combination with cobimetinib in locally advanced or metastatic solid tumors [211,212]. It also did not improve efficacy in combination with paclitaxel in *PIK3CA*, *AKT1*, and *PTEN*-altered HR-positive HER2-negative advanced breast cancer in the IPATunity130 trial [213].

*MSC2363318A* is a CNS penetrant, ATP competitive inhibitor of p70S6K, AKT1, and AKT3 inhibitor which has shown activity in breast, pancreatic, and ovarian cancer cell lines in preclinical studies and was first evaluated in phase I study in patients with advanced malignancies with PI3K/AKT/PTEN pathway alterations, but the best response was stable disease [214]. Currently, no further developments are noted.

*Vevorisertib (ARQ751)* is another novel pan-AKT inhibitor that was investigated in preclinical studies as a single agent or in combination with sorafenib in hepatocellular carcinoma in cirrhotic rat models [215]. It was first evaluated in a phase Ib study as a single agent or in combination with paclitaxel or fulvestrant in *PIK3CA/AKT/PTEN*-mutated advanced solid tumors and demonstrated only modest clinical activity (5% in the monotherapy group and 20% ORR in the vevorisertib–paclitaxel group) [216].

### 5.2. mTOR Inhibitors

mTOR is another downstream substrate of the PI3K signaling pathway. Everolimus and temsirolimus were first-generation mTOR inhibitors that selectively activate mTORC1 activity while second-generation mTOR inhibitors inhibit both mTORC1 and mTORC2 [217].

*Temsirolimus*, mTORC1 inhibitor, was the first mTOR inhibitor to be FDA-approved on 30 May 2007, for the treatment of advanced RCC based on a phase III Global ARCC trial [218,219]. Temsirolimus was compared to interferon alfa in treatment-naive patients with RCC and demonstrated improved OS of 10.9 months in comparison to interferon alfa alone group (7.3%) (HR 0.73, 95% CI 0.58–0.92; *p* = 0.008) and better ORR of 8.6% vs. 4.8% [219]. The most common adverse effects were asthenia, rash, anemia, nausea, and anorexia with the most frequent grade 3 or higher adverse effects being asthenia, anemia, and hyperglycemia [219].

*Everolimus* is another mTORC1 inhibitor that was FDA-approved for multiple indications including RCC, subependymal giant cell astrocytoma associated with tuberous sclerosis, advanced HR-positive breast cancer, progressive pancreatic neuroendocrine tumors (NETs) and nonfunctional gastrointestinal and lung neuroendocrine tumors, as well as adult patients with renal angiomyolipomas and tuberous sclerosis complex [220]. It was first approved on 30 March 2009 for the treatment of advanced kidney cancer after progression on sunitinib or sorafenib based on a phase III RECORD-1 trial where everolimus was compared to the placebo [221,222]. Its approval in pancreatic NETs on 6 May 2011 was based on the phase III RADIANT-3 trial while its approval for gastrointestinal and lung NETs on 26 February 2016 was based on the RADIANT-4 trial where it was compared to placebo due to significant improvement in PFS [223,224]. The most common adverse events were stomatitis, rash, diarrhea, fatigue, and infections [223]. Everolimus plus exemestane in the phase III BOLERO-2 trial in HR-positive, HER2-negative breast cancer patients pretreated with AI demonstrated a PFS benefit of 6.5 months compared to exemestane alone (HR 0.36, 95% CI 0.27–0.47; *p* < 0.001), leading to its FDA approval on 20 July 2012 [225].

*Sapanisertib (MLN0128/TAK-228)* is a pan-mTOR inhibitor that has demonstrated variable degrees of efficacy in clinical trials. Sapanisertib only showed limited activity as a single agent in R/R acute lymphoblastic leukemia and advanced pancreatic NETs, as well as in combination with paclitaxel in recurrent or persistent endometrial cancers [226,227,228]. However, it demonstrated some clinical activity in combination with exemestane or fulvestrant in previously treated postmenopausal women in combination with carboplatin and paclitaxel in mTOR pathway-altered advanced solid tumors, as a single agent in advanced solid tumors as well as in combination with cisplatin in nasopharyngeal carcinoma [40,229,230,231]. Currently, it is being evaluated in locally advanced or metastatic bladder cancer with tuberous sclerosis mutations (NCT03047213) and in combination with paclitaxel in advanced or recurrent ovarian, fallopian tube, or primary peritoneal cancer (NCT03648489).

**Figure 2:**
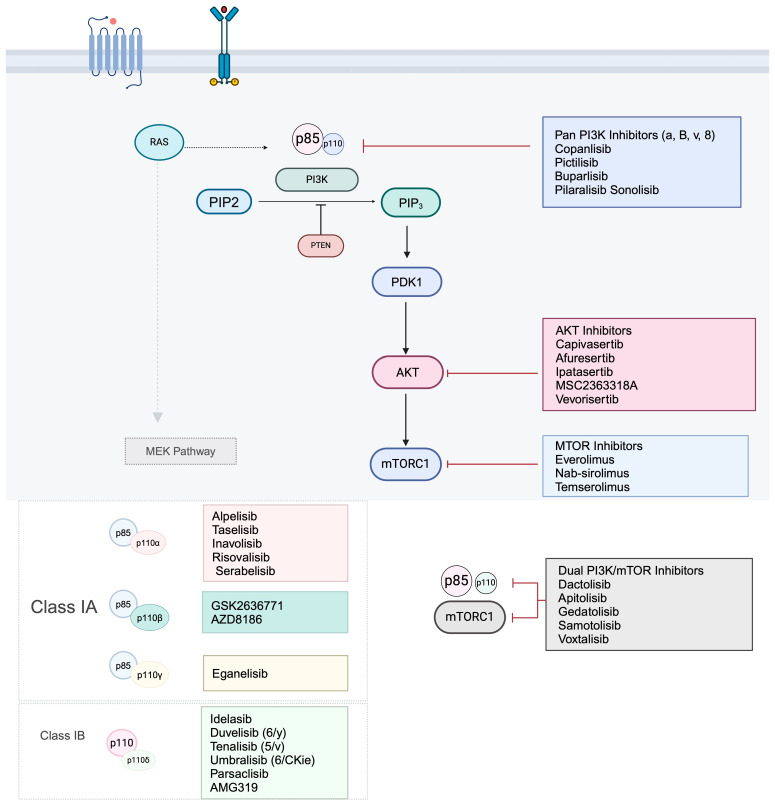
Overview of PI3K/AKT/mTOR target drugs along the PI3K pathway in oncology. PI3K inhibitors can be subclassified into pan PI3K, isoform-specific inhibitors and dual PI3K/mTOR inhibitors. Isoform-specific PI3K inhibitors can be sub-classified into PI3Kβ, PI3Kδ, PI3Kα, and PI3Kδ inhibitors. Created with BioRender.com.

Figure 2 illustrates PI3K/AKT/mTOR target drugs at their specific target sites along the PI3K pathway.

### 5.3. Mechanism and Management of the Most Relevant Toxicity

The systematic toxicities of PI3K inhibitors usually originate from their primary inhibiting mechanisms to other critical cellular processes in addition to the original target, which is the deranged *PIK3CA* gene [232]. Diarrhea (43%), fatigue (30%), nausea (30%), cough (29%), and pyrexia (28%) were the most common adverse effects of idelalisib seen in the indolent NHL trial by Gopal et al. with diarrhea being the most common grade 3 or higher adverse effect. Adverse events leading to the discontinuation of idelalisib were elevated alanine or aspartate aminotransferase levels (4%), colitis (3%), pneumonia and pneumonitis (2% each), diarrhea and neutropenia (2% each). Prednisone and enteric budesonide could be considered in idelalisib-related colitis.

In the DYNAMO trial, the most common adverse effects of duvelisib were gastroenterological-related, such as diarrhea (49%), nausea (29.5%), neutropenia (29%), and fatigue (28%). Colitis was reported in 7.8% and pneumonitis in 4.7% of patients. The discontinuation rate was seen in 31% of patients mostly due to pneumonitis, pneumonia, colitis, diarrhea, and rash while dose interruptions or reductions were seen in 66% of patients mostly due to diarrhea, febrile neutropenia, and increased lipase levels [88]. In the DUO trial, adverse effects were similar with colitis being the most common grade 3 or higher adverse effects (12%) [87]. Umbralisib had similar adverse effects in the UNITY-CLL trial with neutropenia, diarrhea, transaminitis, and colitis [233].

Hyperglycemia was the most common on-target effect of alpelisib and was seen in 63.7% of patients in the SOLAR-1 trial, followed by diarrhea (57.7%), nausea (44.7%), decreased appetite (35.6%), and rash (35.6%). Hyperglycemia was also the most common grade 3 or 4 adverse effects seen in 36.6% of patients, leading to the discontinuation of alpelisib in 6.3% of patients followed by a rash (3.2%) [84]. p110α subunit of PI3K is involved in the regulation of insulin signaling, thus the inhibition of PI3K leads to the inhibition of insulin-dependent signaling, leading to increased glycogen breakdown in the liver and decreased skeletal muscle and adipose tissue glucose uptake. This process leads to hyperglycemia and, at the same time, the insulin feedback pathway causes PI3K/AKT/mTOR pathway activation causing attenuation of the therapeutic response of PI3K inhibitors [234,235]. Hyperglycemia is an important adverse effect to look out for in patients with alpelisib requiring dose reductions, treatment interruptions, and drug discontinuations. Therefore, patients taking alpelisib should be instructed to adjust their diet according to the American diabetic association guidelines and lifestyle modifications to decrease the risk of developing hyperglycemia [236]. In addition, a personalized antihyperglycemic regimen including metformin, sodium–glucose co-transporter 2 inhibitors, and insulin sensitizer pioglitazone could be considered to optimize blood glucose levels [236]. Given the short half-life of alpelisib, blood glucose levels can be normalized in 24 to 72 h after dose interruption and short-term insulin could be used if needed. Alpelisib can be reintroduced at a lower dose if hyperglycemia improves to less than or equal to grade one adverse effect [236]. With the information learned from prior studies, structured interventions to control hyperglycemia have decreased the overall discontinuations of alpelisib due to hyperglycemia in the BYLieve trial [38]. Hyperglycemia was also a common adverse effect of conpalisib, followed by transient hypertension, diarrhea, fatigue, decreased neutrophil count, and fever. Adverse effects leading to drug discontinuations were seen in 15% of patients including non-infectious pneumonitis, lung infection, and hyperglycemia [83].

As mentioned above, common toxicities include gastrointestinal disturbances including diarrhea/colitis, nausea, skin lesions, fatigue, cytopenias, hyperglycemia, hepatotoxicity, hypertension, and pneumonitis [234,235]. However, the most common side effects are dose-related and reversible with a reduction in or discontinuation of treatment [237]. Integrative approaches such as adjusting dosage, time limiting, and choosing suboptimal dose selection are recommended to reduce toxicities. Adverse effects have been the major factor in limiting its utilization in cancer patients.

## 6. Resistance Mechanisms to PI3K Inhibitors

The involvement of the PI3K gene as a targetable oncogene has been commonly detected in different types of solid malignancies [238]. Despite that, the total effectiveness of PI3K inhibitors in cancers is still challenging due to limited target gene engagement from the reactivation of pathways by compensatory mechanisms and crosstalks with other signaling pathways [4,20]. Other mechanisms, such as the gene derangement of the PI3K gene, inactivation of PTEN, which is a negative modulator of the PI3K pathway, and the deregulation of ncRNAs (Non-Coding RNAs), which leads to the over-activation of PI3K signaling pathway, could lead to drug resistance [239]. Primary resistance is due to the initial lack of response to treatment while secondary resistance happens after the initial response to treatment.

### 6.1. Primary Resistance

#### 6.1.1. ESR1 and PTEN Baseline Mutations

Mutation-activating mutations in *ESR1* and *PTEN* have been described as a primary mechanism of resistance to the selective PI3Kα inhibitor alpelisib in combination with AI and HR-positive metastatic breast cancer. Longitudinal analysis of ctDNA (circulating tumor DNA) revealed that nearly half (15/32) of the non-responders had baseline *PTEN* or *ESR1* mutations [240].

#### 6.1.2. MAP2K1, BRAF, and KRAS Activating Mutations

In R/R CLL patients who did not respond to idelalisib, baseline activating mutations in *MAP2K1, BRAF,* and *KRAS* genes were identified in approximately 60% of non-responders. This was determined using baseline whole-exome sequencing. These mutations result in the inability to inhibit ERK phosphorylation [241]. Interestingly, combination therapy with CI-1040 (MEK1/2 inhibitor) and SCH772984 (selective ERK1/2 inhibitor) has been demonstrated to reduce the percentage of divided cells [241,242,243]. These findings were reproducible with the FDA-approved MEK inhibitor trametinib [244].

### 6.2. Secondary Resistance

#### 6.2.1. PIK3CA Acquired Mutations

In a recent publication by Varkartis et al., it was observed that approximately half of the patients with *PIK3CA*-mutated HR-positive, HER2-negative advanced breast cancer treated with PI3Kα inhibitors developed secondary resistance via the PI3K pathway. Liquid biopsies and autopsies were used to assess mutations and alterations, and it was found that a change in the drug-binding site caused a newly acquired PI3K-resistant pathway. However, allosteric PI3K inhibition could overcome this resistance mechanism [245].

#### 6.2.2. Loss of PTEN Function

The increased signaling of PI3K p110β isoform due to the acquired loss of function of PTEN was first clinically described in breast cancer by longitudinal analysis of multiple metastases on an HR-positive breast cancer patient with an initial robust response to alpelisib [240,246].

#### 6.2.3. Insulin Derived PI3K Activation

The Warburg Effect is a canonical principle in cancer cells, characterized by a high energy consumption rate due to the increased consumption of glucose and lactate via aerobic glycolysis instead of oxidative phosphorylation. This phenomenon results in the increased uptake of glucose through aerobic glycolysis and lactate production [247]. The connection between the Warburg effect and the MAPK pathway has been explained through two primary mechanisms: enhanced intracellular glucose transport and insulin resistance. AKT-mediated phosphorylation raises GLUT1 (glucose transporter 1) receptors and reduces GLUT4 membrane translocation, creating a high intracellular environment that is crucial for protein expression and cancer cell survival. After the stimulation of the insulin receptor and insulin-like growth factor receptors (INSR and IGFR), insulin receptor substrate receptor molecules (IRS) are activated. IRS-1 interacts with the catalytic p110 subunit, leading to increased PIP3 and p85 regulatory subunit sequestration, which is associated with increased insulin resistance and can explain the side effects of PIK3CA inhibitors, such as hyperglycemia. IRS-1 stimulation increases GLUT-1 receptor expression and downregulates GLUT4 membrane translocation [248,249,250,251].

A recently published phase 2 clinical trial investigated the effects of hyperglycemia in an orthotopic glioblastoma (GBM) xenograft model by combining antihyperglycemic interventions, such as a ketogenic diet and metformin, with the PI3K inhibitor buparlisib. The results showed that a combination of metformin and PI3K inhibitors improved survival and reduced insulin feedback [252]. Combination therapy enhanced the inhibition of PI3K and identified systemic hyperglycemia as an independent activator of insulin-mediated mechanisms and independent of AKT phosphorylation.

PI3K inhibitors significantly reduced AKT phosphorylation in the control arm but increased the levels of insulin receptor phosphorylation without inhibiting S6 phosphorylation. This suggests that systemic hyperglycemia alone can trigger insulin signaling in GBM. Further studies focusing on optimal glycemic control strategies with PI3K inhibitors may improve the tolerability and outcomes of PI3K inhibitors.

Traditionally, immunotherapy faced limitations in GBM patients due to T cell depletion. However, PI3K inhibitors have emerged as agents capable of increasing the recruitment of microglia and T-cells, thereby presenting a potential avenue for exploring the role of immunotherapy in GBM treatment.

#### 6.2.4. Downstream Secondary Resistance

In a recent paper by Varkartis et al., approximately 50% of acquired resistance to alpelisib was due to *PTEN* loss or the activation of *AKT1* mutation and secondary resistant mutations that alter the PI3K binding pocket, reducing its efficacy [245].

### 6.3. Endocrine-Mediated Resistance

Adaptive mechanisms by the activation of endocrine-mediated factors and signaling upon PI3K inhibition have been described to confer secondary resistance to PI3KIs. Upon PI3K inhibition, ER-positive breast cancer cells can increase ER sensitivity and bypass PI3K by directly activating AKT. A treatment strategy has been established to target ER and PI3K concomitantly by adding anti-estrogen therapy with PI3KIs as in the SOLAR-1 trial [253]. In contrast, in prostate cancer, the inhibition of PI3K combined with anti-androgen therapy results in increased transcription factors mediated by androgen, mainly through the loss of *PTEN* [254,255,256].

### 6.4. Non-Genetic Drug Tolerance

Genetically unaffected cell populations have been shown to activate transcription without genetic stimulation and can survive target inhibition [255,257]. Although these cells possess some secondary resistance mechanisms, they lack the ability to continuously replicate, which is a key characteristic of cancer tumorigenesis. This lack of replication ability is believed to be one of the mechanisms of resistance to PI3K target medication and immunotherapy [258]. Epithelial–mesenchymal transition (EMT) has been proposed as a mechanism by which epithelioid tumors transform into mesenchymal cells. EMT differs from primary resistance in that it involves a nonreplicating state. This phenomenon is thought to be driven by a change in phenotype or cell plasticity and is similar to the protective feature of diapause, which occurs during the blastocyte phase in utero. It is worth noting that this state can be reversed upon drug withdrawal, indicating that it is not driven by genetic mutations that promote drug tolerance [259,260].

## 7. Landscape of Ongoing Investigations, Challenging Issues, and Future Directions

Despite their drawbacks due to toxicities and resistance mechanisms, there are currently several ongoing trials of PI3KIs in various cancers. However, some of the trials have been terminated due to the lack of efficacy or intolerable toxicities. There have been developments of more isoform-selective PI3KIs, dual PI3K/mTOR inhibitors, and allosteric mutant PI3KIs to overcome these drawbacks.

Given the prevalence of PI3K pathway alterations across various tumor types and their extensive preclinical and clinical evidence, the PI3K pathway has major contributions to the development of cancers and clinical implications. Despite the success of PI3KIs and their FDA approvals, further development of drugs in this class was halted due to the limited efficacy across different tumor types and intolerable toxicities. Early PI3KIs evaluated were pan-isoform inhibitors without selectivity to specific PI3K isoform and had limited efficacy. They have intolerable toxicities due to both on-target and off-target activities. However, the next generations of PI3KIs are designed to target specific isoforms, leading to improved activity and better safety profiles. There have also been developments of better PI3KI, including isoform and mutant selective PIK3CA inhibitors with improvement in glucose dysregulation. Another challenging issue with PI3KI is resistance to PI3KI, making PI3KI monotherapy less effective. The loss of *PTEN* mutation is one of the important mechanisms in developing resistance, leading to PIP3 accumulation in the plasma membrane and AKI/mTOR pathway activation [120]. Therefore, targeting other essential players in the AKT/mTOR pathway may alleviate the issues related to drug resistance.

The dysregulation of the PI3K pathway is known to be associated with immune checkpoint inhibitors (ICI) like PD-1/PDL1 inhibitors. PI3K pathway inhibition can cause a reduction in immunosuppressive cytokines and immunosuppressive Treg cells, thus enhancing the activity of ICI. PI3K pathway inhibition in combination with ICI is effective in PTEN-driven murine melanoma models and lung cancer mouse models and currently being investigated in early phase trials [261,262]. 

Further novel investigations of PI3KIs will lead to the evaluation of their role in chimeric antigen receptor T cells (CAR-T). Duvelisib has been shown to inhibit CAR-T induced interleukin-6 (IL-6) production from immature dendritic cells while demonstrating no inhibitory effect on CAR-T, thus minimizing cytokine release syndrome (CRS) related to CAR-T cell therapy in vitro and in vivo, which warrant further evaluation of its utilization in the treatment of CAR-T-associated CRS [263]. In addition, the exposure of CAR-T cells to duvelisib has been demonstrated to improve the effectiveness of CAR-T cells in the elimination of CLL in vitro and vivo studies by enriching CD8+ CAR-T cells [264]. Alpelisib has also been approved for indications other than malignancies. It has been approved by the FDA for adult and pediatric patients with severe manifestations of PIK3CA-related overgrowth spectrum (PROS) syndrome on 5 April 2022 based on the EPIK-P1 trial [265]. Another PI3Kδ inhibitor leniolisib was also approved by the FDA on March 2023 for the treatment of activated PI3Kδ syndrome (APDS), which is an inborn error of immunity in pediatric and adult patients with APDS based on its efficacy in phase III trial [266,267].

Finally, in an analysis of current clinical trials by Davoodi-Moghaddam et al., 68% of current phase III and IV trials of PI3K pathway inhibitors are in solid tumors with RCC and breast cancers being the most prevalent, and lymphoma is the most focused hematological malignancy being investigated. The study suggested that mTOR inhibitors could be effective as a monotherapy or in combination with other therapies in solid tumors while PI3KIs are more effective in hematological malignancies especially when combined with immunotherapy and chemotherapy [268]. Given the intolerable toxicities of PI3KIs, only a few investigational drugs reach phase III trials, and monotherapy use with PI3KIs is not promising given the compensatory mechanisms mentioned above. Therefore, the future directions of these drugs would be their combination with other therapies for more effective response, further investigations for the management of drug toxicities, and further development of novel drugs with fewer toxicities. Table 3 summarizes current ongoing clinical trials of PI3K pathway inhibitors either alone or in combination with other therapies.

## 8. Conclusions

There have been several developments in the PI3K pathway inhibitors in the last decade as we discussed above, and five PI3KIs were FDA-approved. However, there are only two PI3K inhibitors, idelalisib and duvelisib, left in the market for hematological malignancies, and alpelisib for breast cancers. Challenging issues faced with PI3KIs are due to intolerable toxicities requiring dose reductions and resistance mechanisms. In addition, capivasertib has also been recently approved by the FDA in breast cancers and it is the first AKT inhibitor to have FDA approval, paving the way for the development of more AKT inhibitors in the future. There are more ongoing investigations on PI3K pathway inhibitors and their combination therapies with immunotherapies and other targeted therapies, opening avenues for the development of novel and more effective treatments. More research is needed in this field to evaluate the tolerance and optimal dosing of PI3KIs in early-phase clinical trials. There is still hope for the PI3K pathway inhibitors in precision medicine with further evaluations to understand more about their adverse effects, management of toxicities, molecular alterations, and resistance mechanisms.

## Figures and Tables

**Table 1:** Frequency of mutations between *PIK3CA*, *AKT1*, and *PTEN* [23,24,25].

Type of Cancer	*PIK3CA*	*AKT1*	*PTEN*
Uterine Endometrial Carcinoma	66.7	6.63	62.5
Invasive Breast Carcinoma	38.35	7.52	7.69
Cervical Cancer	37.03	3.7	7.07
Anal cancer	35	-	12.5
Penile Cancer	27.27	9.09	18.18
Urothelial Carcinoma	27.07	5.36	7.76
Vaginal cancer	23.81	-	-
Head and Neck Cancer	23.71	2.1	4.86
Colorectal cancer	30.56	2.86	8.09
Bladder Cancer	24.75	5.38	3.89
Ovarian Epithelial Tumor	21.41	-	6.5
Esophageal Carcinoma	19.02	3.3	5.035
Gallbladder carcinoma	12.92	-	-
Meningioma	0.82	12.4	24.18
Lung cancer	44.35	5.36	11.11
Sex cord stromal tumor	-	4.55	-
Prostate cancer	5.41	13.85	49.18
Endometrial carcinoma	5.21	6.29	86.19
Glioblastoma	8.96	2.02	38.38
Pancreatic cancer	3.27	17.43	-

**Table 2:** Previous and current FDA-approved PI3K inhibitors with supporting trials.

PI3K Inhibitor	Trial	Phase	Study Population	Intervention/ Subgroups (Number, n)	ORR %	DOR Month	PFS Month	OS Month	FDA Approval
Copanlisib	Dreyling et al. CHRONOS-1 2017 [83]	II	R/R indolent B-cell lymphoma after two or more lines of therapy including rituximab and alkylating agents	Copanlisib n = 142	59	22.6	11.2	NR	14 September 2017. Withdrawn on 12 November 2023
Alpelisib	Andre et al. SOLAR-1 2019 [84]	III	HR-positive HER2-negative advanced breast cancer patients who have received prior endocrine therapy	Alpelisib+ fulvastrant n = 169 (PIK3CA mutant)	26.6		11	-	24 May 2019
				Fulvestrant n = 172 (PIK3CA mutant)	12.8		5.7	-	
				Alpelisib+ fulvastrant n = 115	-		7.4	-	
				Fulvestrant n = 116	-		5.6	-	
Idelalisib	Furman et al. Study 116 2014 [85]	III	Relapsed CLL	Idelalisib + Rituximab n =110	81	-	NR	NR	23 July 2014
				Rituximab n = 110	13	-	5.5	NR	
	Gopal et al. 2014 [86]	II	R/R indolent NHL with prior rituximab and alkylating agent	Idelalisib n = 125	57	12.5	11	20.3	23 July 2014 in relapsed FL and SLL. Withdrawn on 19 January 2022
Duvelisib	Flinn et al. DUO 2018 [87]	III	R/R CLL, SLL, and FL after at least two prior lines of treatment	Duvelisib n = 160	74	-	13.3	NR	24 September 2018, withdrawn in September 2022 for FL
				Ofatumumab n = 159	45	-	9.9	NR	
	Flinn et al. DYNAMO 2019 [88]	II	R/R NHL including FL, SLL, and MZL including prior treatment with rituximab and chemotherapy or radioimmunotherapy	Duvelisib n = 129	47	10	9.5	28.9	
Umbralisib	Fowler et al. UNITY-NHL [89]	II	R/R MZL, FL, or SLL with a prior line of treatment including anti-CD20 regimen	Umbralisib n = 208	47	-	-	-	5 February 2021, withdrawn due to safety on 1 June 2022
				MZL	49.3	NR	NR	-	
				FL	45.3	11.1	10.6	-	
				SLL	50.0	18.3	20.9	-	

**Table 3:** Selected current ongoing trials of PI3K inhibitors [151].

PI3K Inhibitors	Trial	Indications/Study Population	Phase	Status	Experimental Combinations
**Pan-PI3K Inhibitors**					
Copanlisib (BAY 80-6946)	NCT03789240	Untreated Follicular Lymphoma	II	A, NR	+ Rituximab
	NCT03884998	Richter’s Transformation or Transformed Indolent NHL	I	R	+ Nivolumab
	NCT04939272	Relapsed or Refractory MCL	I/II	R	+ Venetoclax
	NCT04895579	Unresectable stage III NSCLC	I	R	+ Durvalumab
	NCT04263584	Previously Untreated DLBCL	II	R	+ Rituximab, cyclophosphamide, doxorubicin, vincristine, prednisolone (RCHOP)
	NCT03842228	Advanced Solid Tumors with Actionable Mutations in PTEN or PIK3CA hot spot mutations	I	R	+ Olaparib + MEDI4736 (Durvalumab)
Buparlisib (NVP-BKM120, AN2025)	NCT04338399	Recurrent or Metastatic HNSCC (BURAN)	III	A, NR	+ Paclitaxel vs. paclitaxel alone
	NCT04975958	Locally Advanced/Metastatic Tumors	I	R	+ Atezolizumab
**Isoform-Specific inhibitors**					
**PI3Kα inhibitors**					
Alpelisib (NVP-BLY719)	NCT05038735	HR+ HER2− Advanced Breast cancer after CDK 4/6 Inhibitor and Aromatase Inhibitor (EPIK-B5)	III	R	+ Fulvestrant
	NCT05143229	Metastatic or Locally recurrent HER2 Negative Breast Cancer	I	R	+ Sacituzumab govitecan
	NCT04762979	HR+, HER2− PIK3CA-Mutant Breast Cancer Progressed on Endocrine Therapy	II	R	+ Endocrine therapy (Fulvestrant, Aromatase inhibitor)
	NCT04251533	Advanced Triple Negative Breast Cancer Who Carry Either a PIK3CA Mutation or Have PTEN Loss (EPIK-B3)	III	A, NR	+ Nab-paclitaxel
	NCT05063786	PIK3CA-Mutated Previously Treated HER2+ Advanced Breast Cancer (ALPHABET)	III	R	+ Trastuzumab +/− Fulvestrant vs. Trastuzumab + chemotherapy
	NCT05230810	PIK3CA-Mutant HER2+ Metastatic Breast Cancer previously treated with two FDA-approved anti-HER2 therapy	I/II	R	+ Tucatinib
	NCT04997902	Adult Recurrent/Metastatic HNSCC	I/II	R	+Tipifarnib
	NCT04729387	Platinum-resistant/Refractory, High-grade Serous Ovarian Cancer, with no Germline BRCA Mutation	III	A, NR	+ Olaparib
Inavolisib (GDC-0077, RG-6114)	NCT05646862	HR+ HER2− PIK3CA-Mutated Locally Advanced or Metastatic Breast Cancer Post CDK4/6 inhibitor and Endocrine Combination Therapy	III	R	+ Fulvastrant vs. Alpelisib + Fulvastrant
	NCT05306041	HER2+, HR+ PIK3CA-Mutated Early Breast Cancer as Neoadjuvant Therapy	II	R	+ PHESGO (Trastuzumab/Pertuzumab), Endocrine therapy vs. PHESGO and Endocrine therapy
	NCT04191499	PIK3CA mutant HR+, HER2- Locally Advanced or Metastatic Breast Cancer (INAVO120)	II/III	R	+ Palbociclib + Fulvestrant vs. Placebo + Palbociclib + Fulvestrant
	NCT04486352	Recurrent or Persistent Endometrial Cancer	II	R	+ Atezolizumab, includes other targeted agents
	NCT04849364	Circulating Tumor DNA positive with genomic target PI3K pathway in residual Stage I-III Triple Negative Breast Cancer (as adjuvant)	II	R	+ Capecitabine +/− pembrolizumab
	NCT04931342	Biomarker Derived Therapies in Persistent or Recurrent Rare Epithelial Ovarian Tumors (BOUQUET)—PIK3CA altered tumors	II	R	+ Palbociclib, or + Palbociclib + Letrozole or + Olaparib or + Giredestrant or + Bevacizumab
	NCT05894239	PIK3CA-Mutated HER2+ Locally Advanced or Metastatic Breast Cancer (maintenance therapy)	III	R	+ PHESGO vs. Placebo + PHESGO
	NCT04589845	Tumor-Agnostic Precision Immunooncology and Somatic Targeting—PIK3CA multiple mutant-positive advanced solid tumors (TAPISTRY)	II	R	N/A
Risovalisib (CYH-33)	NCT04586335	Advanced Solid Tumors including Ovarian, Prostate, Endometrial, and Breast Cancers with DDR gene mutations and/or PIK3CA mutations	I	R	+ Olaparib
	NCT05043922	Recurrent/Persistent Ovary Clear Cell Carcinoma	II	R	None
MEN 1611 (CHS5132799)	NCT04495621	PIK3CA-Mutated Metastatic Colorectal Cancer (C-PRECISE-01)	I/II	A, NR	+ Cetuximab
	NCT05810870	Previously treated PIK3CA/PTEN altered HER2 negative Advanced Breast Cancer (SABINA)	II	R	+ Eribulin
Serabelisib (TAK-117/ MLN 1117)	NCT05300048	Advanced Solid Tumors with PI3CA mutation and PTEN loss of function mutation	I	R	+ Insulin Suppressing Diet +/− Nab-paclitaxel
**PI3Kβ inhibitors**					
GSK2636771	NCT04439188	Advanced Cancers with complete loss of PTEN Expression (MATCH-Subprotocol P)	II	A, NR	
	NCT04439149	Advanced Cancers with PTEN mutation or deletion (MATCH-Subprotocol N)	II	A, NR	
	NCT03131908	Metastatic Melanoma and PTEN loss	I/II	A, NR	+ Pembrolizumab
**PI3Kδ inhibitors**					
Idelalisib (CAL-101/ GS-1001)	NCT05725200	Previously Treated Metastatic Colorectal Cancer—Individualized treatment (EVIDENT)	II	R	N/A
	NCT03257722	Metastatic or recurrent NSCLC who stopped responding to immunotherapy	I/II	R	+ Pembrolizumab
	NCT04666038	Previously Treated CLL/SLL (BRUIN CLL-321)	III	R	+ Rituximab or bendamustine + Rituximab Vs BTK inhibitor LOXO-305
Duvelisib (IPI-145) PI3Kγδ inhibitors	NCT05044039	NHL/Acute Lymphocytic Leukemia following Chimeric Antigen Receptor T-cell Therapy	I	R	None
	NCT04890236	To Enhance Immune Profiles of T Cells in Patients with Recurrent or Refractory DLBCL (DEEP T cells Study)	I	R	Duvelisib exposure before Tisagenlecleucel CAR-T cell manufacturing
	NCT04688658	Advanced Unresectable Melanoma progressed on anti-PD1 therapy	I/II	R	+ Nivolumab
	NCT05010005	Relapsed and Refractory T-cell and NK Cell Lymphoma	I	R	+ Ruxolitinib
	NCT05065866	Lymphoid Malignancy (Hodgkin, Non-Hodgkin, Lymphocytic Leukemia, Myeloma)	I	R	+ BMS986345 (azacitidine)
	NCT03534323	Relapsed or Refractory CLL or SLL or Ritcher Syndrome	I/II	R	+ Venetoclax
	NCT05057247	Recurrent/Metastatic HNSCC	II	R	+ Docetaxel
	NCT04331119	As Maintenance after Autologous Stem Cell Transplant in T-cell Lymphoma	II	R	
	NCT04803201	Previously Untreated CD30 Negative Peripheral T-Cell Lymphomas	II	R	+ cyclophosphamide, doxorubicin, vincristine, etoposide, and prednisone
	NCT05044039	NHL/Acute Lymphocytic Leukemia following Chimeric Antigen Receptor T-cell Therapy	I	R	
Parsaclisib (INCB-50465, IBI376)	NCT04774068	Relapsed or Refractory T-Cell and NK-Cell Lymphomas	I	R	+ Romidepsin
	NCT03235544	Relapsed or Refractory MCL Previously Treated with or without a Bruton’s Tyrosine Kinase Inhibitor (CITADEL-205)	II	A, NR	
	NCT04298879	Relapsed or Refractory Marginal Zone Lymphoma (CITADEL-204)	II	A, NR	
	NCT04809467	Relapsed or Refractory NHL or Chronic Lymphocytic Leukemia (topMIND)	I/II	A, NR	+Tafasitamab
	NCT04551066	Myelofibrosis (LIMBER-313)	III	A, NR	+ Ruxolitinib
	NCT04551053	Myelofibrosis (LIMBER-304) with suboptimal response to Ruxolitinib	III	A, NR	+ Ruxolitinib
	NCT04323956	Newly Diagnosed High-Risk DLBCL	I	A, NR	+ Rituximab, Cyclophosphamide, Doxorubicin, Vincristine, and Prednisone +/− Polatuzumab-vedotin (PaR-CHOP)
Linperlisib (YY-20394)	NCT05274997	Relapsed or Refractory Peripheral T/NK Cell Lymphoma	II	R	None
	NCT05676710	Relapsed or Refractory Large Granular T Lymphocytic Leukemia	I	R	None
Umbralisib (TGR-1202)	NCT03269669	Relapsed or Refractory Grade I-IIIa Follicular Lymphoma	I/II	R	In combination with obinutuzumab
Zandelisib (PWT-143/ME-401)	NCT04517435	Newly Diagnosed DLBCL	I/II	R	+ Rituximab, Cyclophosphamide, Doxorubicin, Vincristine, Prednisone (R-CHOP)
**PI3Kγ inhibitors**					
Eganelisib (IPI-549)	NCT03961698	Treatment Naïve Triple-Negative Breast Cancer or Renal Cell Carcinoma (MARIO-3)	I	A, NR	+ Atezolizumab, Nab-paclitaxel, Bevacizumab
	NCT03795610	Resectable locally Advanced HPV+ and HPV− HNSCC prior to surgery	II	R	None
**Dual PI3K/mTOR inhibitors**					
Gedatolisib (PKI-587)	NCT03065062	Advanced squamous cell lung, pancreatic, head and neck, and other solid tumors	I	R	+ Palbociclib
	NCT05501886	Advanced or metastatic HR+/HER2− breast cancer who progressed on CDK4/6 therapy (VIKTORIA-1)	III	R	+ Fulvestrant +/− Palbociclib
Paxalisib (GDC-0084, RG7666)	NCT04906096	Recurrent or Refractory Primary CNS Lymphoma	II	R	
	NCT05183204	Newly Diagnosed and Recurrent Glioblastoma	II	R	+ Metformin + Ketogenic Diet
**AKT inhibitors**					
Capivasertib	NCT04493853	Metastatic Hormone Sensitive Prostate Cancer and PTEN Deficiency (CAPItello-281)	III	R	+ Abiraterone + androgen deprivation therapy (ADT) vs. placebo + abiratero + ADT
	NCT05348577	Metastatic Castration Resistant Prostate Cancer (CAPltello-280)	III	R	+ Docetaxel vs. Placebo + Docetaxel
Afuresertib	NCT05383482	Selected Solid Tumors that are resistant to prior anti-PD1/PD-L1	I/II	R	+ Sintilimab + chemotherapy (nab-paclitaxel or docetaxel)
Ipatasertib	NCT04253561	Advanced HER2+ PI3KCA-mutant Breast Cancer Patients (IPATHER)	I	R	+ Trastuzumab and Pertuzumab
	NCT04467801	Metastatic NSCLC Patients who failed 1st line Immunotherapy (Ipat-Lung)	II	R	
	NCT05172245	Stage III-IVB Head and Neck Cancer	II	R	+ chemotherapy + radiation
**mTOR inhibitors**					
Everolimus	NCT03008408	Advanced or Recurrent Endometrial Carcinoma	II	R	+ Letrozole +/− Ribociclib
	NCT05012371	Metastatic Renal Cell Cancer as second or third Line	II	R	+ Lenvatinib vs. Cabozantinib
**Others:**	Target				
LOXO-783	NCT05307705	Breast Cancer or other solid tumors with a PIK3CA H1047R Mutation	I	R	+/− Fulvestrant, Imlunestrant, Abemaciclib, Anastrozole, Exemestane, or Letrozole, Paclitaxel
RLY-2608	NCT05216432	HR+ HER2− Advanced Breast Cancer	I	R	+/− Fulvestrant
STX-478	NCT05768139	Advanced Solid Tumors	I	R	+/− Fulvestrant

R = recruiting; A, NR = active, non-recruiting.

## Data Availability

Not applicable.
